# Implicit bias and experience influence overall but not relative trustworthiness judgment of other-race faces

**DOI:** 10.1038/s41598-024-66705-7

**Published:** 2024-07-11

**Authors:** Olivia S. Cheung, Nathan J. Quimpo, James Smoley

**Affiliations:** 1 Department of Psychology, Science Division, New York University Abu Dhabi, Abu Dhabi, UAE; 2 Center for Brain and Health, NYUAD Research Institute, New York University Abu Dhabi, Abu Dhabi, UAE

**Keywords:** First impressions, Face, Trustworthiness, Implicit bias, Other-race effect, Human behaviour, Psychology

## Abstract

Impressions of trustworthiness are formed quickly from faces. To what extent are these impressions shared among observers of the same or different races? Although high consensus of trustworthiness evaluation has been consistently reported, recent studies suggested substantial individual differences. For instance, negative implicit racial bias and low contact experience towards individuals of the other race have been shown to be related to low trustworthiness judgments for other-race faces. This pre-registered study further examined the effects of implicit social bias and experience on trustworthiness judgments of other-race faces. A relatively large sample of White (N = 338) and Black (N = 299) participants completed three tasks: a trustworthiness rating task of faces, a race implicit association test, and a questionnaire of experience. Each participant rated trustworthiness of 100 White faces and 100 Black faces. We found that the overall trustworthiness ratings for other-race faces were influenced by both implicit bias and experience with individuals of the other-race. Nonetheless, when comparing to the own-race baseline ratings, high correlations were observed for the relative differences in trustworthiness ratings of other-race faces for participants with varied levels of implicit bias and experience. These results suggest differential impact of social concepts (e.g., implicit bias, experience) vs. instinct (e.g., decision of approach-vs-avoid) on trustworthiness impressions, as revealed by overall vs. relative ratings on other-race faces.

## Introduction

First impressions of various personality inferences can be reliably extracted at a glance of a face^1,2^. Despite the lack of evidence that first impressions accurately predict actual personality traits^3–5^, such impressions are difficult to overcome^6^. Because of the important function of trust in initial and subsequent social interactions for assessing the target individual’s intention, rapid trustworthiness judgments based on faces have shown to be consequential in real-world situations such as resource sharing or sentencing decisions^7–10^. Extensive research has examined behavioral and neural effects of trustworthiness judgments^11,12^. Critically, trustworthiness judgment from faces is found to be highly consistent among individual raters, as certain faces are consistently rated to be more trustworthy than others^12^.

The high consensus in trustworthiness judgment could be due to either evolutionary importance of such judgments, or learning of social stereotypes for the personality traits from the environment, although these two accounts are not mutually exclusive^13–15^. However, a potential limitation in the literature is that the majority of findings on trustworthiness judgments were primarily based on data from White participants perceiving White faces, which limits the generalizability of the findings to non-White samples and poses questions about whether trustworthiness judgments are consistent across participant races and face races^15–17^.

Indeed, mixed findings have been reported on cultural and individual differences in trustworthiness evaluation of own- and other-race faces. When comparing the trustworthiness ratings between White and Black faces, Black male faces are sometimes perceived as more positive and sometimes as more negative, compared with White male faces^17–20^. These findings revealed possible in-group biases or attempts to overcorrect the biases^18,21–23^. More importantly, however, considerable individual differences or environmental influences have been found to account for the variability of trustworthiness judgments of faces^14^, more than differences in cultural background^20,23–28^. Nonetheless, substantial similarities in judgment of trustworthiness and other personality traits remain^24,25,27^.

To what extent do the characteristics of the perceivers contribute to the individual differences observed in face evaluation^29^? Much research has shown that the processing of other-race faces is often worse than that of own-race faces^30–33^. It has been suggested that the ‘other-race’ effect might be due to the perceivers’ lack of expertise or motivation to process the other-race faces at the individual level compared with the category level (e.g., race)^34–38^. Although early findings did not find evidence for explicit prejudice attitudes on other-race face processing (see meta-analysis^32^), the ‘other-race’ effect has more recently been shown to be influenced by implicit social bias towards individuals of other races in several aspects of face processing, including perceptual processing and threat and trustworthiness evaluations on other-race faces^9,39–47^. There has also been evidence of implicit bias effects on own-race face processing^48,49^.

This study aimed to further characterize the effects of implicit bias and experience on trustworthiness judgment of other-race faces. These factors were chosen because of the prevalence of their effects on other-race face perception. In the literature, different approaches have been suggested to measure the similarities and differences in first impression judgments among individuals^27,50^. In this study, White and Black participants completed three tasks: they first rated the perceived trustworthiness of 100 White faces and 100 Black faces, then completed an Implicit Association Test (IAT) with White and Black faces, and finally completed a brief questionnaire on social contact experience with White and Black people. We tested two questions on the influences of implicit bias and experience on trustworthiness judgment of other-race faces. The first question focused on the overall trustworthiness judgments of other-race faces or own-race faces among participants, replicating previous findings; the second question instead focused on relative trustworthiness judgments among individual faces within each race by examining whether certain faces were consistently rated to be more trustworthy-looking than others by participants with different levels of implicit bias and experience. To our knowledge, this is the first study to examine the effects of implicit bias and experience on both overall and relative trustworthiness ratings of other-race faces.

Both sets of analyses were initially conducted separately for White and Black participants but with the same approaches. First, to investigate the effects of implicit bias and experience on overall trustworthiness judgment of other-race faces, regression analyses were conducted using the averaged ratings of the 100 faces of either own- or other-race for each participant. Apart from the two main predictors of implicit bias and experience, the overall trustworthiness rating of own-race faces of each participant was also included in the analyses to increase the explained variance in the data, as individual differences in trust might generalize across faces of different races. More importantly, we expected to replicate and extend previous findings^9^ that participants with negative implicit bias and limited other-race experience would rate other-race faces more negatively than participants with positive implicit bias and extensive other-race experience. We also expected that similar results would be observed for both White and Black participants.

Second, to examine how implicit bias and experience may affect the relative trustworthiness ratings of individual faces across faces, we examined whether certain faces were consistently rated to be more trustworthy than other faces within each race by different subgroups of participants with varied levels of implicit bias or other-race experience. Following previous procedures^42^, this analysis correlated ratings of each of the individual faces from various subgroups of participants with the baseline ratings. To establish the appropriate relative trustworthiness baseline of own-race faces, the ratings of each of the 100 White faces were averaged across all White participants as the baseline for White faces, whereas the ratings of each of the 100 Black faces were averaged across all Black participants as the baseline for Black faces. Then four subgroups for White participants and four subgroups for Black participants were created based on their implicit bias towards the other-race (positive vs. negative) or their experience with members of the other race (high vs. low). For each of the 100 Black faces, the ratings for each face were averaged within each of the four White participant subgroups and were then correlated with the own-race baseline ratings from Black participants; for each of the 100 White faces, the ratings for each face was averaged within each of the four Black participant subgroups and were then correlated with the own-race baseline ratings from White participants. We expected that participants with positive implicit bias or extensive experience towards the other race would correlate with the own-race baseline ratings, because they would be just as capable or motivated as participants of the same race to individuate the faces. In contrast, participants with negative implicit negative bias or limited experience might show low correlations in their relative ratings of the individual other-race faces compared with the own-race baseline ratings, potentially because they primarily categorize other-race faces at the race level and not the individual level^36^. Alternatively, if implicit bias or experience do not affect how participants perceive the relative trustworthiness of other-race individuals, similar relative trustworthiness ratings would be observed among participants of different levels of implicit bias or experience towards other-race individuals, compared with the own-race baseline.

## Method

### Transparency

The study was pre-registered on https://osf.io/hd6ts.

### Participants

A total of 401 White participants and 397 Black participants who were United States nationals participated online via the Prolific platform (www.prolific.io). The study was approved by the New York University Abu Dhabi Institutional Review Board. All participants gave informed written consent prior to the experiment. All methods were performed in accordance with the relevant guidelines and regulations.

All participants reported to be between 18 and 40 years old, fluent in English, and have normal or corrected to normal vision. All White participants and 332 Black participants were recruited with the 100% approval rate criterion on the platform, following the pre-registered plan. For the rest of the 65 Black participants, the approval rate was subsequently changed to a minimum of 90% approval rate because of the relatively slow recruitment progress. Note that the data quality was comparable between the two sub-samples of Black participants, as the proportion of excluded data was comparable between the 100% approval rate group (24.4%) and the group with a minimum of 90% approval rate (26.2%).

The pre-registered exclusion criteria aimed to ensure data quality by removing data that was likely impacted by participants not properly following task instructions or getting distracted from the tasks. Specifically, data of participants were excluded if any of the criteria below were met: (1) incomplete data files; (2) extreme outliers with response times below 200 ms or over 5000 ms for more than 10% of the trials in either the trustworthiness rating task or the Implicit Association Test; (3) over 10 consecutive trials with the same response in the trustworthiness rating task; (4) below 0.5 standard deviation in the trustworthiness ratings, which suggested that participants did not perform the task seriously and constantly responded with the same or neighboring keys.

The final analyses included data from 338 White participants and 299 Black participants. In a post-hoc sensitivity analysis, the final sample size of approximately 300 participants from each race was shown to have sufficient power of > 0.99 to obtain the fixed effects tested, based on a medium effect size. Of this final sample, there were 276 White female, 55 White male, 201 Black female, and 79 Black male participants who provided gender information. For the data included in the final analyses, trials with response times below 200 ms or over 3 standard deviations from each participant’s average response times were excluded from the analyses.

### Stimuli

The trustworthiness rating task used a total of 100 White faces and 100 Black faces, with 50 male and 50 female faces from each race, from the Chicago Face Database^51^. The faces were selected based on the trustworthiness norming data from the database^51^, with half of the faces from each race and gender rated as trustworthy (White faces: *M* = 3.78, *SE* = 0.034; Black faces: *M* = 3.96, *SE* = 0.029), and the other half of the faces from each race and gender rated as untrustworthy (White faces *M* = 2.91, *SE* = 0.031; Black faces: *M* = 3.01, *SE* = 0.035). Based on the ratings of the selected faces, a two-way ANOVA with the factors Trustworthiness and Face race revealed a significant main effect of Trustworthiness, *F*_1,196_ = 808.80, *p* < 0.001,* η*_*p*_^*2*^ = 0.805, confirming that the trustworthy faces were rated higher in trustworthiness than the untrustworthy faces. Moreover, the significant main effect Face race, *F*_1,196_ = 18.05, *p* < 0.001, *η*_*p*_^*2*^ = 0.084, showed that the Black faces were rated more trustworthy than the White faces. There was no significant interaction between Trustworthiness and Face race, *F*_1,196_ = 1.74, *p* = 0.189, *η*_*p*_^*2*^ = 0.009. All faces in the trustworthiness rating task had a neutral expression and all faces were shown in frontal view. The face images were approximately 250 pixels in height and 190 pixels in width.

The Implicit Association Test (IAT) used a total of ten White faces and ten Black faces, with five male and five female faces from each race, from the MR2 face database^52^. The selected faces had a neutral expression and were shown in frontal view. The face images were approximately 250 pixels in height and 190 pixels in width. The IAT also used a total of ten positive words (savior, kindness, loyal, pleasure, laughter, happy, excellent, trust, friend, honest) and a ten negative words (disaster, hatred, traitor, destroy, terrible, brutal, despise, abuse, useless, toxic). According to Bellezza et al.^53^, these words were significantly different in ratings for positive vs. negative meaning (7.85 out of 9 for positive vs. 2.15 out of 9 for negative, *t*_18_ = 28.90, *p* < 0.001).

The experience questionnaire was adapted from the individuating experience and social contact questionnaires from Walker and Hewstone^38^, as we updated the race information appropriate for the present study. There was a total of 20 items. For individuating experience, a set of 5 items were used for each of the two races, White or Black, to measure how often participants engaged in activities with individuals of each race. An example item was “I have looked after or helped a White (Black) friend when someone was causing them trouble or being mean to them” with the scale: *Very often, Quite often, Sometimes, Hardly ever,* and *Never.* For social contact experience, another set of 5 items were used to measure the interactions with people of each of the two races, White or Black. An example item was “How many White (Black) people do you know very well?” with answer options: *Up to 2, Up to 5, Up to 8, Up to 12, and More than 12*.

### Procedure

Each participant completed three tasks in the same order: the trustworthiness rating task, the IAT, and the experience questionnaire. We believe that it was important for participants to first complete the trustworthiness rating task, since the subsequent tasks might raise participants’ awareness that either implicit bias or experience with own vs. other-race individuals was being examined, which might influence their responses. Because the experience questionnaire asked about relatively objective questions and should be least likely affected by prior tasks, it was administered at the end. All tasks were programmed and presented using Testable (www.testable.org)^54^. The entire study lasted approximately 30 min.

#### Trustworthiness ratings

The trustworthiness rating task assessed first impressions of the faces. Participants rated the perceived trustworthiness of each of the 200 face images (“How trustworthy is this person?”), using a 7-point scale (1: not at all; 7 very much). On each trial, a fixation was first presented for 500 ms, followed by a face image that was presented for 100 ms. Participants were given unlimited time to respond using a key press. The presentation order of all the White and Black face images was randomized for each participant. Cronbach’s $\alpha$ of the trustworthiness rating tasks for either White or Black faces was high for both White and Black participants (all ≥ 0.97). Prior to the actual study, participants were given 20 practice trials which used a separate set of White and Black faces.

#### Implicit Association Test (IAT)

The race IAT followed the procedure of Greenwald et al.^55,56^. On each trial, participants categorized whether a face was either White or Black, or whether a word had a positive or negative meaning. The response times in categorizing White vs. Black faces and positive vs. negative words with specific pairings of the response keys were measured.

The IAT included a total of five blocks of trials^55^. In Block 1, a White or Black face was randomly presented on each of the 40 trials. Participants were asked to categorize each face by race and respond on the keyboard by pressing either a key for White faces or another key for Black faces. In Block 2, a positive or negative word was randomly presented on each of the 40 trials. Participants were asked to categorize each word by valence and respond on the keyboard by pressing either a key for positive words or another key for negative words. In Block 3, either a face (White or Black) or a word (positive or negative) was randomly presented on each of the 120 trials. Participants categorized each face or word using the assigned response keys from Blocks 1 and 2. For half of the participants, one response key was assigned for positive words and White faces, and another response key for negative words and Black faces. For the other half of the participants, the response key mapping in Block 3 was with a response key assigned for positive words and Black faces, and another key for negative words and White faces. In Block 4, the procedure was identical to Block 1, except that the response keys for the different face races were switched. In Block 5, the procedure was identical to Block 3, except that the response keys for the faces were identical to the assigned keys in Block 4.

The main analysis of implicit biases focused on Blocks 3 and 5, following the improved algorithm from Greenwald et al.^56^. In Blocks 3 and 5, the first 40 trials were considered ‘practice’ trials, and the remaining 80 trials were considered ‘test’ trials. The practice and test trials were first analyzed separately, and the scores were then averaged to form the final scores. The *D* score reveals implicit biases by the differential performance between the response key mappings between White-positive/Black-negative and Black-positive/White-negative blocks. A positive *D* score indicated a positive implicit bias towards White people, whereas a negative *D* score indicated a positive bias towards Black people. To produce a *D* score for each participant, response times for the correct trials were first calculated, after outliers removed (< 250 ms or > 10 s). Response times for the incorrect trials were replaced with the mean response times of the corresponding block and two times of the standard deviations of the correct response times of the block.

For the reliability of the race IAT in the present study, Cronbach’s $\alpha$ of the *D* scores were 0.70 for White participants and 0.74 for Black participants. The correlations between the response times in the practice and test trials that participants responded correctly for the White-positive/Black-negative block were *r* = 0.730, *p* < 0.001 for White participants and *r* = 0.726, *p* < 0.001 for Black participants. The correlations between response times in the practice and test trials for the White-negative/Black positive block were *r* = 0.790, *p* < 0.001 for White participants and *r* = 0.737, *p* < 0.001 for Black participants.

#### Experience questionnaires

For the experience questionnaire, participants reported the frequency of interactions with White and Black people. The scores of the 5 items on each of the individuating and social contact questionnaires were averaged to obtain the final scores. Cronbach’s $\alpha$ for the questionnaires on individuating and social contact experience with White people was 0.90 and 0.85 for White participants, and 0.92 and 0.92 for Black participants, respectively. Cronbach’s $\alpha$ for the questionnaires on individuating and social contact experience with Black people was 0.92 and 0.89 for White participants, and 0.91 and 0.85 for Black participants, respectively. We found that the ratings between the individuating and social contact scales were positively correlated in both our samples: For White participants, the correlations between the two experience scores were *r* = 0.655, *p* < 0.001 for Black people, and *r* = 0.508, *p* < 0.001 for White people. For Black participants, the correlations between the two experience scores were *r* = 0.560, *p* < 0.001 for Black people, and *r* = 0.686, *p* < 0.001 for White people. Following prior research^45^, we averaged the experience scores of the two scales for subsequent analyses.

## Results

Following the pre-registered analysis plan, we conducted the main analyses reported below, including the regression analyses on overall trustworthiness ratings of own- and other-race faces, and the correlation analyses on relative trustworthiness ratings of individual faces of the other race. Additionally, we first reported the descriptive and inferential statistics on overall trustworthiness ratings, implicit bias, and experience of White and Black participants.

### Descriptive and inferential statistics on trustworthiness ratings, implicit bias, and experience

**Figure 1:**
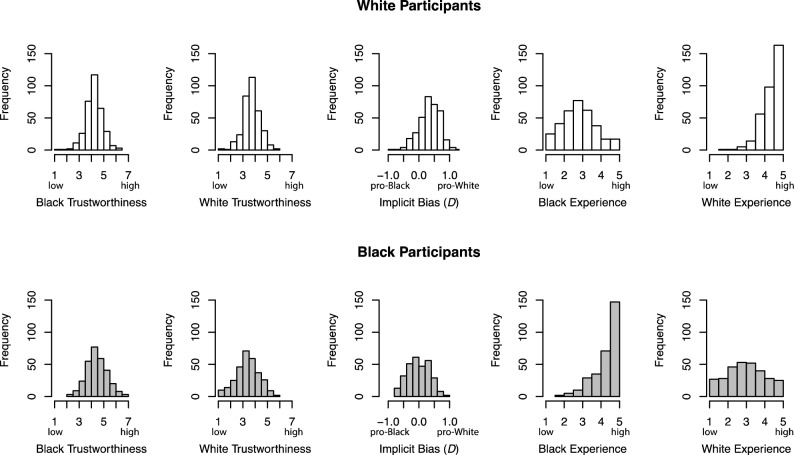
Distributions of trustworthiness ratings of Black and White faces, implicit bias, and experience scores of Black and White people of the White (top panel) and Black (bottom panel) samples.

Table 1 shows the descriptive statistics of trustworthiness ratings, implicit bias, and experience scores of White and Black participants. Figure 1 illustrates the distributions of these measures.

**Table 1:** Descriptive statistics on trustworthiness ratings, implicit bias, and experience of the White and Black samples.

	Mean	SD	Max	Min
White participants				
Trustworthiness ratings for Black faces	4.23	0.684	6.35	1.09
Trustworthiness ratings for White faces	3.71	0.675	5.88	1.23
Implicit bias (*D*)	0.344	0.334	1.20	-0.871
Experience with Black individuals	2.86	0.903	5.00	1.80
Experience with White individuals	4.45	0.546	5.00	1.80
Black participants				
Trustworthiness ratings for Black faces	4.43	0.818	6.58	2.28
Trustworthiness ratings for White faces	3.37	0.919	5.71	1.08
Implicit bias (*D*)	− 0.0126	0.355	0.865	− 0.755
Experience with Black individuals	4.35	0.706	5.00	1.60
Experience with White individuals	3.01	1.04	5.00	1.00

**Figure 2:**
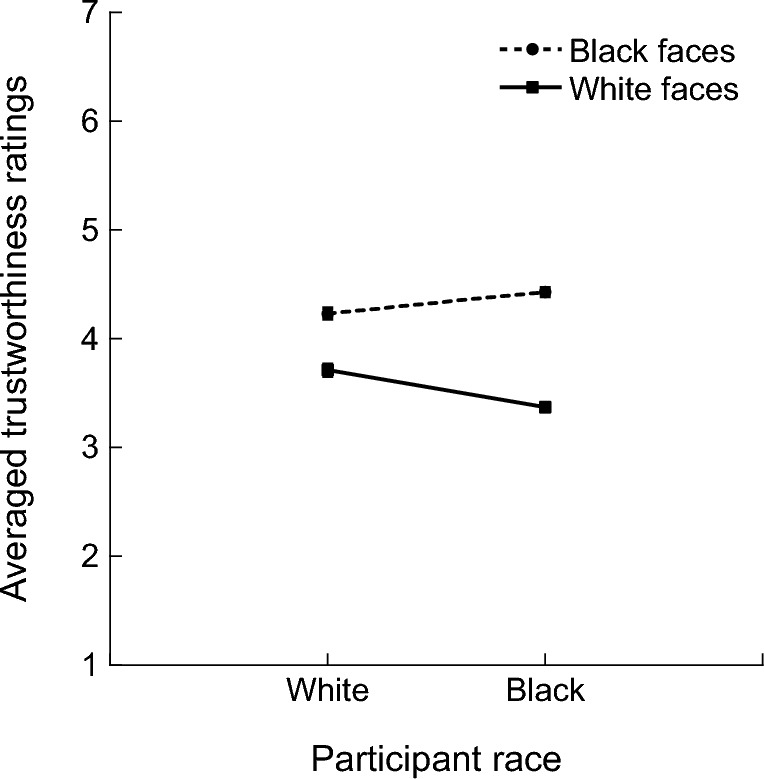
Overall trustworthiness ratings of White faces and Black faces by White participants and Black participants. Error bars represent standard errors.

For the overall trustworthiness ratings (Fig. 2), a two-way ANOVA with a between-subjects factor Participant race (White or Black) and a within-subjects factor Face race (White or Black) revealed no significant main effect of Participant race, *F*_1,635_ = 1.89, *p* = 0.169, ${\eta }_{p}^{2}=0.003$, but a significant main effect of Face race, *F*_1,635_ = 439.0, *p* < 0.001, ${\eta }_{p}^{2}=0.409$, with higher overall trustworthiness ratings for Black faces than White faces. There was also a significant interaction between Participant race and Face race, *F*_1,635_ = 50.8, *p* < 0.001, ${\eta }_{p}^{2}=0.074$, with Black participants showing a larger difference in the trustworthiness ratings between Black faces and White faces, compared with White participants. Nonetheless, follow-up tests showed that higher overall trustworthiness ratings for Black faces than White faces were observed for both Black participants (*t* = 19.27, *p*_tukey_ < 0.001) and White participants (*t* = 10.09, *p*_tukey_ < 0.001).

For implicit bias, there was a range of positive and negative implicit biases among White and Black participants (Table 1; Fig. 1). To examine whether either of the participant groups showed an overall positive vs. negative bias towards the other group, two 2-tailed one-sample *t*-tests against 0 (no bias^57^) revealed that White participants showed an overall negative bias towards Black people (*t*_337_ = 19.0, *p* < 0.001, *d* = 1.03), whereas Black participants did not show an overall bias towards White people (*t*_298_ = 0.539, *p* = 0.539, *d* = − 0.0355).

For experience (Table 1; Fig. 1), we examined whether the White participants and the Black participants had more experience with people of their own race than people of the other race. Indeed, White participants reported having less experience with Black than White people (*t*_337_ = − 28.9, *p* < 0.001, *d* = − 1.57); Black participants reported having more experience with Black than White people (*t*_298_ = 18.60, *p* < 0.001, *d* = 1.08).

### Regression analyses on overall trustworthiness ratings for other-race faces

Table 2 reports the correlations among the dependent measures and the predictors. Table 3 presents the regression results on overall trustworthiness ratings for other-race faces, separately for White and Black participants. The models with the four predictors, trustworthiness ratings of own-race faces, implicit bias, experience with other-race people, and experience with own-race people, explained significant variance in trustworthiness ratings for other-race faces both for White participants, *R*^*2*^ = 0.194, *p* < 0.001, *adjusted R*^*2*^ = 0.183, and for Black participants, *R*^*2*^ = 0.292, *p* < 0.001, *adjusted R*^*2*^ = 0.288. Note that the analyses including participants and stimuli as random factors using linear mixed models essentially showed identical results.

**Table 2:** Correlations among the dependent measures and predictors. *p < 0.05, **p < 0.001.

	1	2	3	4	5
White participants					
1—Trustworthiness ratings for Black faces	–				
2—Trustworthiness ratings for White faces	0.498**	–			
3—Implicit bias	− 0.136*	0.091	–		
4—Experience with Black people	0.111*	0.001	− 0.092	–	
5—Experience with White people	− 0.019	0.077	0.110*	0.093	–
Black participants					
1—Trustworthiness ratings for Black faces	–				
2—Trustworthiness ratings for White faces	0.083	–			
3—Implicit bias	− 0.099	0.223**	–		
4—Experience with Black people	0.280**	− 0.248**	− 0.162*	–	
5—Experience with White people	0.040	0.280**	0.081	0.021	–

**Table 3:** Results of the multiple regression analyses on the averaged trustworthiness ratings of other-race faces by White and Black participants.

Predictor	$\beta$	*B*	*SE*	*t*	*p*
White participants					
Ratings of own-race faces	0.517	0.524	0.047	11.13	< 0.001
Implicit bias	− 0.168	− 0.345	0.096	− 3.60	< 0.001
Experience with other-race people	0.0997	0.0755	0.035	2.14	0.033
Experience with own-race people	− 0.050	− 0.063	0.059	− 1.07	0.285
Black participants					
Ratings of own-race faces	0.165	0.186	0.061	3.02	< 0.001
Implicit bias	0.174	0.449	0.138	3.25	0.001
Experience with other-race people	0.265	0.235	0.047	5.04	< 0.001
Experience with own-race people	-0.271	-0.353	0.072	-4.92	< 0.001

For both White and Black participants, overall trustworthiness ratings for other-race faces were predicted by overall trustworthiness ratings for own-race faces (White participants: $\beta =0.517$, *p* < 0.001; Black participants: $\beta =0.165$, *p* < 0.001), suggesting that participants who generally judged own-race faces to be more trustworthy also judged other-race faces to be more trustworthy, compared with the participants who judged own-race faces to be less trustworthy. These results suggest that the individual differences of White and Black participants in evaluating trustworthiness based on faces were consistent between the different races of faces.

Importantly, implicit bias also similarly influenced trustworthiness ratings for other-race faces for both White and Black participants. For White participants, implicit bias negatively predicted overall trustworthiness ratings for other-race faces, $\beta =-0.168$, *p* < 0.001, revealing that White participants who had stronger positive bias toward White people judged Black faces to be less trustworthy, compared with White participants who had stronger positive bias toward Black people. In contrast, for Black participants, implicit bias positively predicted trustworthiness ratings for other-race faces, $\beta =0.174$, *p* = 0.001. Specifically, Black participants who had stronger positive bias toward White people judged White faces to be more trustworthy, compared with Black participants who had stronger positive bias toward Black people.

Apart from implicit bias, the effect of experience with other-race people was also significant in both White and Black participants (White participants: $\beta$ = 0.517, *p* < 0.001; Black participants: $\beta$ = 0.165, *p* < 0.001), as participants who reported more experience with people of the other race judged the other-race faces to be more trustworthy, compared with participants who reported less experience with people of the other race. The effect of experience with own-race people was not significant in White participants ($\beta$ = − 0.050, *p* = 0.285). Perhaps surprisingly, the effect was significant in Black participants ($\beta$ = − 0.271, *p* < 0.001), suggesting that Black participants who reported more experience with Black people judged White faces to be less trustworthy, compared with Black participants who reported less experience with Black people.

### Regression analyses on overall trustworthiness ratings for own-race faces

For completeness, we also explored how various factors might influence the trustworthiness ratings for own-race faces, although it was expected that the effects would not necessarily be the same as those for other-race faces. Table 4 presents the regression results on overall trustworthiness ratings for own-race faces, separately for White and Black participants. The models with the four predictors explained significant variance in the trustworthiness ratings of own-race faces both for White participants, *R*^*2*^ = 0.281, *p* < 0.001, *adjusted R*^*2*^ = 0.272, and for Black participants, *R*^*2*^ = 0.110, *p* < 0.001, *adjusted R*^*2*^ = 0.0983.

**Table 4:** Results of the multiple regression analyses on the averaged trustworthiness ratings of own-race faces by White and Black participants.

Predictor	$\beta$	*B*	*SE*	*t*	*p*
White participants					
Ratings of other-race faces	0.525	0.518	0.047	11.13	< 0.001
Implicit bias	0.150	0.303	0.096	3.16	0.002
Experience with own-race people	0.076	0.093	0.058	1.61	0.109
Experience with other-race people	− 0.050	− 0.038	0.035	− 1.07	0.287
Black participants					
Ratings of other-race faces	0.182	0.162	0.054	3.02	0.003
Implicit bias	− 0.089	− 0.203	0.131	− 1.56	0.121
Experience with own-race people	0.311	0.360	0.067	5.41	< 0.001
Experience with other-race people	-0.011	-0.008	0.046	-0.18	0.855

Nonetheless, of the four predictors, only the trustworthiness ratings for other-race faces consistently predicted trustworthiness ratings of own-race faces for both White and Black participants (White participants: $\beta$ = 0.525, *p* < 0.001; Black participants: $\beta$ = 0.182, *p* = 0.003). Similarly in the previous analysis on trustworthiness ratings on other-race faces, participants who judged other-race faces to be more trustworthy also judged own-race faces to be trustworthy, compared with those who judged other-race faces to be less trustworthy, suggesting that the individual differences in trustworthiness judgments exist, regardless of the race of the faces.

For White participants, implicit bias also positively predicted trustworthiness ratings of own-race faces, $\beta$ = 0.150, *p* = 0.002, revealing that White participants with higher positive bias towards White people rated own-race faces more trustworthy than White participants with lower positive bias towards White people. For Black participants, experience with own-race people positively predicted trustworthiness ratings of own-race faces, $\beta$ = 0.311, *p* < 0.001, suggesting that Black participants who reported more experience with Black people rated own-race faces more trustworthy than Black participants who reported lower experience with Black people. Unsurprisingly, experience with other-race people did not predict trustworthiness ratings of own-race faces in either White participants ($\beta$ = − 0.050, *p* = 0.287) or Black participants ($\beta$ = − 0.011, *p* = 0.855).

### Correlation analyses investigating the effects of implicit bias and experience of other-race people on relative trustworthiness ratings of individual faces of the other race

To examine the relative trustworthiness judgments of individual other-race faces between participants with different levels of implicit bias and experience with other-race people, the averaged ratings of each of the 100 faces of each race (e.g., Black faces) from the same participant race (e.g., Black participants) were used as the baseline, and the ratings were correlated with the averaged ratings from several subgroups of participants of the other race (e.g., White participants). To maximize the differences between the subgroups based on implicit bias and experience with other-race people, we used data from the participants who had the most positive bias toward White people and the participants who had the most positive bias toward Black people from each of the participant groups (N = 50 in each group). For experience with other-race people, data from the participants with highest and lowest experience were also used (N = 47–53 as some participants had identical scores). The sample size of approximately 50 participants was based on previous studies that showed highly reliable results with a similar sample size (e.g., between 10 and 40 participants)^42^. Note that in the selected sub-groups, there was relatively limited overlap between the low experience groups and the negative bias groups, or between the high experience groups and the positive bias groups (about 15–18% overlap). The descriptive statistics of the selected groups are reported in Tables 5 and 6.

**Table 5:** Descriptive statistics on trustworthiness ratings, implicit bias and experience of the White and Black samples with high vs. low level of experience with Black or White people.

White participants	High black experience		Low black experience	
	Mean	SD	Mean	SD
Trustworthiness ratings for Black faces	4.243	0.744	4.058	0.842
Trustworthiness ratings for White faces	3.817	0.676	3.680	0.651
Implicit bias (*D*)	0.322	0.353	0.384	0.342
Experience with Black individuals	4.377	0.903	1.487	0.245
Experience with White individuals	4.740	0.335	4.628	0.601

Black participants	High white experience		Low white experience	
	Mean	SD	Mean	SD
Trustworthiness ratings for Black faces	4.427	0.942	4.445	0.831
Trustworthiness ratings for White faces	3.707	0.787	2.921	1.000
Implicit bias (*D*)	0.043	0.380	− 0.011	0.318
Experience with Black individuals	4.475	0.679	4.290	0.820
Experience with White individuals	4.536	0.340	1.468	0.300

**Table 6:** Descriptive statistics on trustworthiness ratings, implicit bias and experience of the White and Black subsamples with positive vs. negative biases towards Black or White people.

White participants	Pro-black implicit bias		Pro-white implicit bias	
	Mean	SD	Mean	SD
Trustworthiness ratings for Black faces	4.418	0.640	4.165	0.790
Trustworthiness ratings for White faces	3.509	0.754	3.737	0.804
Implicit bias (*D*)	− 0.218	0.168	0.827	0.134
Experience with Black individuals	3.020	0.916	2.864	0.980
Experience with White individuals	4.298	0.596	4.580	0.523

Black participants	Pro-black implicit bias		Pro-white implicit bias	
	Mean	SD	Mean	SD
Trustworthiness ratings for Black faces	4.585	0.854	4.471	0.801
Trustworthiness ratings for White faces	3.036	0.882	3.691	0.885
Implicit bias (*D*)	− 0.547	0.095	0.504	0.123
Experience with Black individuals	4.470	0.701	4.174	0.842
Experience with White individuals	2.940	1.016	3.166	1.045

**Figure 3:**
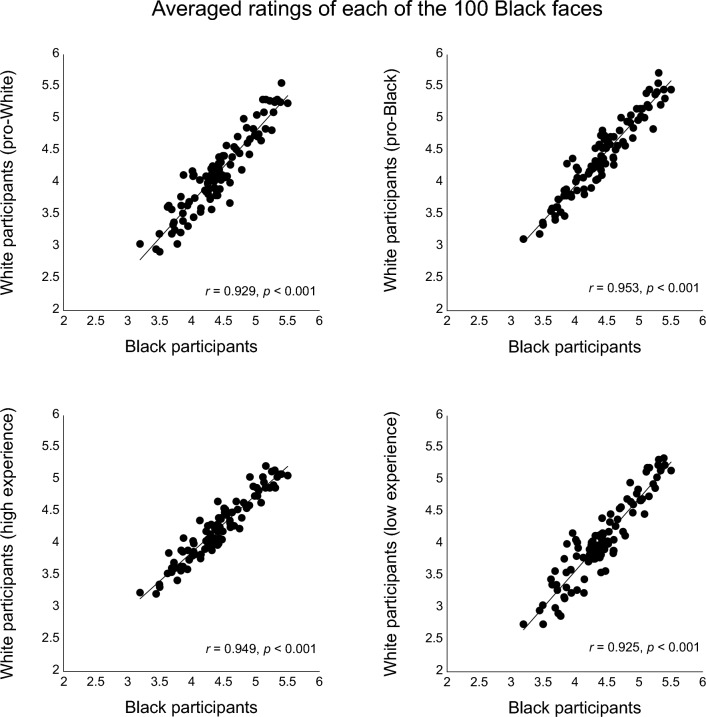
Scatterplots (with best-fitting regression lines) showing the relationship between trustworthiness ratings of the 100 Black faces from all Black participants (own-race baseline) vs. from four subgroups of White participants: (1) with positive implicit bias toward White people (top right), (2) with positive implicit bias toward Black people (top left), (3) with high level of experience with Black people (bottom left), and with low level of experience with Black people (bottom right).

The trustworthiness ratings of the individual faces were highly correlated among all groups. For the 100 Black faces (Fig. 3), the ratings from each of the White participant groups (with positive bias toward White people, with positive bias toward Black people, with high level of experience with Black people, or with low level of experience with Black people) all positively and strongly correlated with the baseline ratings from the Black participant group (*r*’s > 0.92, *p*’s < 0.001).

**Figure 4:**
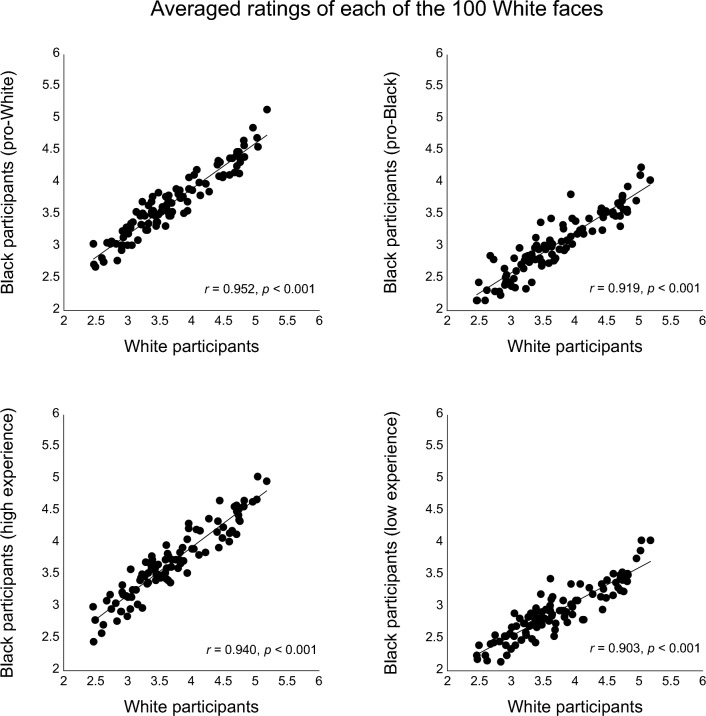
Scatterplots (with best-fitting regression lines) showing the relationship between the trustworthiness ratings of the 100 White faces from all White participants (baseline) vs. the trustworthiness ratings from four subgroups of Black participants: (1) with positive implicit bias toward White people (top left), (2) with positive implicit bias toward Black people (top right), (3) with high level of experience with White people (bottom left), and (4) with low level of experience with White people (bottom right).

Similarly for the 100 White faces (Fig. 4), the ratings from each of the Black participant groups (i.e., positive bias towards White people, with positive bias toward Black people, with high level of experience with White people, or with low experience with White people) also positively and strongly correlated with the baseline ratings from the White participant group (*r*’s > 0.90, *p*’s < 0.001).

## Discussion

The goal of the present study was to examine how perceived trustworthiness for briefly presented other-race faces was influenced by implicit bias and experience towards the other race. Specifically, we tested whether the effects of implicit bias and experience on overall trustworthiness ratings averaged across individual faces of a race, as opposed to relative ratings among individual faces of a race, might reveal different sources of influences on trustworthiness perception. Using a relatively large sample of White participants and Black participants rating both White and Black faces that were presented very briefly, we found that implicit bias and experience influenced overall but not relative trustworthiness judgments of other-race faces. These results suggest that perceiver characteristics of White and Black participants, specifically implicit bias and experience, has an impact on some but not all aspects of perceived trustworthiness of other-race faces.

First of all, the general difference in the trustworthiness ratings for the White and Black faces found in this study replicated the previously collected trustworthiness ratings by the database^51^. While the selected Black faces might indeed be more trustworthy looking than the selected White faces, our results might also suggest participant differences. For instance, more positive ratings from White participants for Black faces than White faces had also been observed in previous studies^18,20,28^, which might potentially be due to demand characteristics in attempt to overcorrect any potential social undesirability^17^. For Black participants, it might not only reflect demand characteristics but also an in-group bias to perceive own-race faces to be more trustworthy than other-race faces. Nonetheless, it is important to note that the overall difference between Black and White faces unlikely influenced the main conclusions of implicit bias and experience on trustworthiness of other-race faces, because similar result patterns of implicit bias and other-race experience were observed for both races of faces.

### Effects of implicit bias, experience, and own-race trustworthiness: overall trustworthiness ratings of other-race faces

In this study, White participants on average showed a pro-White bias, whereas Black participants did not show an overall bias towards either race. With regards to experience with other-race people, both White and Black participants generally reported to have more extensive contact experience with their respective own-race than other-race people.

Nonetheless, it is important to note that in both the White and Black participant samples, there was a wide range of individual differences for overall trustworthiness ratings on both White and Black faces, and for the implicit bias and the experience scores. More critically, the regression results suggest that trustworthiness perception of both White and Black participants is similarly affected by three main predictors: implicit bias towards the other race, experience with other-race people, and the degree of trustworthiness for own-race faces.

Replicating previous findings^9^, our results showed an influence of implicit bias—White participants who had negative implicit bias towards Black people rated Black faces to be less trustworthy than White participants who had positive implicit bias towards Black people. Likewise, Black participants also showed similar effects of implicit bias and experience when evaluating trustworthiness of White faces: Black participants with negative implicit bias towards White people rated White faces to be less trustworthy than Black participants with positive implicit bias towards or more experience with White people.

Moreover, we found an impact of experience with individuals of a race on trustworthiness ratings. White participants who had less experience with Black individuals also rated Black faces to be less trustworthy than White participants who had more experience with Black people^18^, whereas Black participants who had limited experience with White people also rated White faces to be less trustworthy than Black participants who had more experience with White people. Furthermore, our results also showed that both White and Black participants who provided high overall trustworthiness ratings for own-race faces also did so for other-race faces, suggesting individual differences in the overall level of trust in other individuals generalizes across races.

Together, these results reveal three important sources of individual differences in trustworthiness perception of other-race faces—implicit bias towards the other race, experience with other-race people, and own-race trustworthiness ratings—which are robust and consistent for both White and Black participants. The similar effects found for both White and Black participants suggest that these are general attributes of trustworthiness perception. These results highlight the cultural similarities among White and Black participants, and the individual differences among participants within each racial group on overall trustworthiness perception of other-race faces.

### Consistency of relative trustworthiness ratings of other-race faces

Although implicit bias towards the other race and experience with people of the other race were found to affect overall trustworthiness ratings for other-race faces, both factors did not appear to affect the way participants evaluate the relative differences in trustworthiness of the individual target faces of either race. In this study, a range of trustworthy-looking and untrustworthy-looking faces were selected, and the trustworthiness of each set of faces (i.e., White or Black) provided by participants of the same race revealed a high degree of variability in relative trustworthiness among individual faces of each face set. Notably, our correlation results revealed that the consistency of the relative trustworthiness ratings among other-race faces were comparable between both White and Black participants with different levels of implicit bias or experience with the other race, compared with the own-race ratings. Therefore, these factors only modulate the overall baseline level of trust, instead of drastically altering how initial trustworthiness is processed.

Perception of trustworthiness in individual faces might depend on whether the faces are processed at the individual level or at the category level. Other-race faces are more often processed at the category level (e.g., White, Black) and not at the individual level^36^, compared with own-race faces, potentially due to the lack of expertise or motivation to individuate the other-race faces^58,59^. Since positive bias and extensive experience tend to improve processing and recognition performance of other-race faces^34,37,41,46^ but see^45^, it may not be surprising that consensus in trustworthiness judgments on individual faces was observed between same-race participants and other-race participants with positive bias and extensive experience with the other race. Nonetheless, our results showed that even participants with negative bias or limited experience with the other race showed a similar degree of consensus, suggesting that implicit bias or experience might not restrict one’s ability to perceive the relative differences in trustworthiness of other-race individuals. Because the same pattern of results was observed for both White and Black participants, it is likely that the sensitivity to relative differences among individual faces is highly consistent regardless of the race of the target faces, the race of the participants, or other characteristics of the participants, such as implicit bias and experience towards the other race. These findings provide new insights to constrain theories on the perception of trustworthiness.

### Individual differences in overall judgments vs. consensus in relative judgments of other-race faces

There is no doubt of the complexity in understanding first impressions^27,50^. Previous studies suggested that social evaluation depends on three components: characteristics of the perceiver, characteristics of the target, and the interaction between these perceiver characteristics and target characteristics^19,29^. Specifically, perceiver characteristics are likely more influential in the judgment than target characteristics^19^. Our results show that implicit bias and experience are two perceiver characteristics that affect judgments of White and Black participants similarly. The present study demonstrates that different sources of influence on trustworthiness judgments may be revealed by either overall or relative ratings. On the one hand, similar effects of implicit bas and experience with the other race on overall ratings were observed for both White and Black participants. On the other hand, the relative differences among faces were comparable across participants with varied levels of implicit bias or experience with the other race.

Why does overall trustworthiness vary but relative trustworthiness remains relatively stable across perceivers with different characteristics? Individual differences in trustworthiness judgment are readily observed^14^. Although there is no biological or genetic basis for grouping people according to their race^60^, it is possible that implicit bias and experience toward the other race influence evaluation of other-race faces from learned social concepts or stereotypes about in-group vs. out-group^13,61,62^. Such learned social concepts likely bias trustworthiness ratings for other-race faces in one direction, depending on whether the content of the stereotype is positive or negative. In contrast, trustworthiness perception is critical as a survival instinct, in order for the perceiver to determine in an instant whether to approach or avoid another individual. To achieve such intuitions, trustworthy inferences are likely based on an overgeneralization of facial cues resembling emotional expression^63,64^, which may be at least somewhat universal. Indeed, first impressions of other target characteristics, such as likeability, are also found to be quite comparable for White and Black faces for participants of the two races^28^. In this regard, similar first impressions might be formed regardless perceiver characteristics, at least across White and Black participants for White and Black faces.

### Limitations and future directions

Because only two races of faces were included in this study, participants might have realized the purpose of the study and attempt to provide socially desirable responses, such as higher trustworthiness ratings for Black faces than White faces. However, the general difference in overall trustworthiness ratings between Black and White faces did not affect the main conclusions drawn here on the influences of implicit bias and experience on overall trustworthiness ratings, primarily because similarly effects were observed for other-race and own-race faces, instead of faces of a specific race. Nonetheless, it might be possible to minimize potential demand characteristics by including faces from several races^9^ but see^18^, or using a between-subjects design^17^. In any case, future research should consider including multiple races of faces and participants to further examine the generalization and complexity of how various cultural similarities and differences influence the trustworthiness perception of other-race faces.

Although this study shows evidence that trustworthiness perception of individual faces can generalize across face race, discrimination of trustworthiness of the faces may utilize different aspects of visual information. For instance, it has been shown that participants relied more on low spatial frequency content than high spatial frequency content for own-race than other-race faces^65^, especially in participants who had positive own-race bias (i.e., White participants with pro-White bias^66^). Future research should further examine how similar or different aspects of visual information might be used for the evaluation of individual faces, and whether the processing might be influenced by factors such as implicit bias and experience of individual perceivers.

Last but not least, several findings have suggested gender differences in trust^67–69^. As this study revealed the influences of implicit bias and other-race experience on overall but not relative trustworthiness perception of other-race faces, future studies may also consider whether these influences might interact with both the gender of the perceiver and of the faces.

## Conclusion

In sum, the present study showed that implicit bias and experience impact overall trustworthiness judgments of other-race faces, revealing in-group preferences for both White and Black participants. However, observers with varied levels of implicit bias or experience with the other race make highly similar trustworthiness judgments on individual faces, presumably because the capacity to reliably perceive relative trustworthiness among individual faces is instinctive.

## Data Availability

The dataset and analysis files are available on https://osf.io/7up4f/.
